# The profile of Black South African men diagnosed with prostate cancer in the Free State, South Africa

**DOI:** 10.4102/safp.v65i1.5553

**Published:** 2023-01-10

**Authors:** Matthew O.A. Benedict, Wilhelm J. Steinberg, Frederik M. Claassen, Nathaniel Mofolo

**Affiliations:** 1 Department of Family Medicine, Faculty of Health Sciences, School of Clinical Medicine, University of the Free State, Bloemfontein, South Africa; 2 Department of Urology, Faculty of Health Sciences, School of Clinical Medicine, University of the Free State, Bloemfontein, South Africa; 3 Faculty of Health Sciences, School of Clinical Medicine, University of the Free State, Bloemfontein, South Africa

**Keywords:** prostate cancer, Black men, African men, risk factors, social determinants, disease stage and grade

## Abstract

**Background:**

Prostate cancer (PCa) ranks high in terms of morbidity and mortality, especially in Africa. Prostate-specific antigen (PSA) screening remains a practical method of screening for and thereby detecting PCa early, especially among African men who are more negatively affected. Modifiable risk factors for PCa are mostly behavioural and lifestyle. Understanding community-specific determinants is important when developing health promotion interventions.

**Objective:**

This study aimed to determine the profile of African men with PCa in the Free State, South Africa.

**Method:**

A cross-sectional descriptive study was conducted using case record information and self-administered questionnaires among 341 African men with PCa attending the oncology and urology clinics of a tertiary hospital.

**Result:**

Participants’ median age at diagnosis was 66 years. Only 76 (22.3%) participants had ever heard of PCa prior to being diagnosed with the disease, 36 (47.4%) of whom had ever had screening performed. The majority (*n* = 298, 87.4%) were symptomatic; < 50% sought medical help within six months. At diagnosis, 133 (39.0%) men presented with stage T3 or T4 disease, 75 (22.0%) with metastatic disease and 84 (24.6%) with Gleason score ≥ 8. Factors associated with advanced and high-grade disease included smoking, decreased sunlight exposure and physical activity, relatively increased ingestion of dairy products and red meat. Factors associated with early stage and low-grade disease included relatively increased ingestion of fruits, vegetables and fish.

**Conclusion:**

Advanced and high-grade PCa disease is not uncommon among men ≥ 60 years in this study setting. Certain modifiable risk factors associated with advanced disease were established in this study. The majority had lower urinary tract symptoms (LUTS) prior to PCa diagnosis, but they were of poor health-seeking behaviour. Although there seems not to be a systematic delay in the definitive diagnosis and initiation of treatment for PCa, there is a need to improve on health education and awareness in the study setting.

## Introduction

Globally, cancer is a major health burden and is on an upward trend. Estimates from the Global Cancer Observatory of the International Agency for Research on Cancer showed an incidence and mortality of 18.1 million and 9.6 million, respectively, in 2018. These figures increased to 19.3 million and about 10 million, respectively, in 2020.^1,2^ Prostate cancer (PCa) ranks the second most frequent cancer diagnosis and the fifth leading cause of death among men worldwide. Its global incidence and mortality for 2018 were 1.3 million and 360 000, respectively. These figures increased to 1.4 million and 375 000, respectively, in 2020.^1,2^ The impact is greater in Africa and low- and middle-income countries (LMICs) because of genetic, socio-economic and sociocultural factors.^3,4^ In South Africa, PCa is the most common cancer among men,^5^ and there has been an increase in PCa incidence rate from 29 per 100 000 men in 2007^6^ to 68 per 100 000 men in 2018.^7^ Prostate cancer accounts for about 13% of male deaths from cancer in South Africa.^8^ Prostate cancer in Black South African men is more likely to be hereditary than in other racial groups; hence, they are disproportionately affected.^9^ The South African government, through the National Development Plan 2030, sets out nine long-term health goals, one of which is to ‘significantly reduce prevalence of non-communicable diseases’.^10^

Prostate-specific antigen (PSA) screening for PCa, although controversial because of the associated false-positive results, overdiagnosis, overtreatment and the related complications,^11^ remains a practical method of early detection, early treatment and prevention of metastatic disease and complication,^12^ especially in Africa where there is higher mortality compared with other regions of the world.^13^

According to the United States (US) Preventive Task Force, there is a likelihood for a decreased mortality from PCa in men aged 55–69 years with PSA screening; there is currently no benefit shown in screening men above 70 years of age.^11,14^ In contrast, the South African PCa diagnostic and treatment guidelines (SAPCDTGs)^15^ recommend PSA testing for men with a life expectancy of more than 10 years and with any of the following criteria: (1) Black Africans ≥ 40 years and those with family history of prostate or breast cancer in a first-degree relative, (2) men of other races ≥ 45 years and (3) men with history of lower urinary tract symptoms (LUTS) and clinical suspicion of PCa, regardless of age group.

According to an unpublished work by Myburg 2016 et al.^16^ from the urology department of Universitas Academic Hospital, Bloemfontein, Free State, South Africa, African men, compared with their European counterparts, had PCa associated with worse prognosis (i.e. Gleason score ≥ 8), higher mean PSA levels and more locally advanced stage, at presentation. These results are corroborated by previous studies on racial disparities in PCa presentation.^17,18,19,20,21,22^

Another unpublished audit of PCa cases from January 2019 to July 2019 at the same department revealed that curative treatment was possible in only about 10% (38 out of 366) of the cases, whereas 77% of PCa cases are localised, according to the National Cancer Institute, United States.^23^

Risk factors that have been associated with PCa are either nonmodifiable (e.g. increasing age, ethnicity, genetic factors and family history) or modifiable, for example diet (increased intake of saturated animal fat and red meat, coffee consumption, lower intake of fruits, vegetables and vitamins), smoking, obesity, physical inactivity, infections and environmental exposure to chemicals or ionising radiation. Modifiable risk factors are mostly behavioural and lifestyle factors.^9,14^

In a review article to establish the determinants of PCa risk, stage at diagnosis and survival among African-American men, poor socio-economic status, lack of social support and network and poor access to healthcare services were associated with unfavourable outcome.^24^ In a United States study, it was concluded that separate PCa screening guidelines might be beneficial to the African-American population.^25^ More empirical and evidence-based studies may therefore be necessary among African men, who are more susceptible to developing PCa.^26,27,28^

Unlike the nonmodifiable risk factors, some of the modifiable risk factors for PCa are community specific. Environmental exposure to chemicals such as pesticides, herbicides, chromium and cadmium is an important risk factor among African men in the Free State province, as many are employed in the agricultural and mining industries.^29^ An understanding of community-specific determinants of this disease and the risk factors associated with the stage and grade at diagnosis is an important step towards the development of relevant health promotion interventions.^30^ Although population screening for PCa is currently not supported, once a patient deemed to belong in the high-risk category attends a healthcare facility, he should be considered for screening through a shared decision process.^31^

### Aim and objectives

This study aimed to determine the profile of African men with PCa in the Free State province, South Africa. The primary objective was to identify their sociodemographic and background characteristics, common clinical features, risk factors for PCa, stage and grade of PCa disease. The secondary objective was to determine factors associated with the stage and grade of the disease at diagnosis.

## Materials and methods

### Study design

This was a cross-sectional descriptive study describing the characteristic features of African men diagnosed with PCa in the Free State province of South Africa.

### Target population and sampling

The target population was African men seen and diagnosed histologically with PCa at the urology and oncology units of Universitas Academic Hospital, a teaching hospital in the Free State province of South Africa. For the purpose of this study, ‘African men’ are defined as self-identified indigenous Black South African men.

Using convenience sampling, all African men diagnosed histologically with PCa attending the urology and oncology clinics for follow-up over a period of just over six months (21 January 2021 to 31 July 2021) were included in the study. All patients of non-black races (including mixed race patients) were excluded from the study. Also, five participants were excluded; three were nonconsenting while the other two were too weak to participate. In total, 341 participants were included in the study.

### Measurement, data collection and the questionnaire

Data were collected using a self-administered survey questionnaire. Parameters in the questionnaire were adapted from similar studies^17,32,33^ that aimed to understand the profile of patients with PCa. Patients diagnosed with PCa and attending the urology and oncology clinics for follow-up visits were requested to complete the questionnaire. Adequate time was allowed for participants to read and understand the study information and consider their consent prior to participation. Upon their consent, the researcher administered the questionnaires to them. Completed questionnaires were collected the same day and immediately kept secured.

The questionnaire consisted of two sections, A and B. Section A enquired about participants’ sociodemographic and background details, that is, age, cultural group, level of education, occupation (including mine workers, exposure to pesticides and herbicides), relationship status and residential area.

The following data pertained to the participants’ PCa, that is, events leading to PCa diagnosis, PCa symptoms reported, duration between onset of symptoms and presentation, previous PCa screening, year of diagnosis, duration between urology appointment and PCa diagnosis, duration between PCa diagnosis and treatment initiation, PCa history among first-degree relatives, medical comorbidities, prior history of sexually transmitted diseases (STDs), cancer stage at diagnosis, Gleason score, recalled history (from 20 years old) of physical activities, diet, body size, exposure to sunlight and smoking.

The questionnaire was translated into the languages spoken most commonly in the area, that is, Sesotho and IsiZulu.^34^

A trained research assistant fluent in these local languages helped with further clarification of questions to the participants who required such help.

Information unknown to the participants, such as cancer stage, Gleason score and other technical features, were obtained from Meditech (Universitas Adacemic Hospital electronic clinical record system) and recorded on the questionnaire by the researcher.

### Steps taken to minimise measurement error

Case and data duplication was prevented by using a colour-coding system where the front cover of a participant’s case record was marked by the researcher for easy identification of those who had already participated.

### Content validity of questionnaire

The questionnaire was adapted from previous similar peer-reviewed studies.^17,32,33^ A Health Sciences Faculty evaluation committee consisting of consultant family physicians, a urologist, medical educators, a professional nurse and a biostatistician subjected the questionnaire to review and approval.

### Pilot study

The questionnaire (using the applicable language version) was pretested on the first 10 participants (in succession) to ensure that the questions were balanced and correctly constructed and that the crucial information would be obtained. The 10 piloted questionnaires were included in the study since no significant changes arose from the pilot study.

### Data analysis

The data were analysed by the first author, using SAS version 9.3 (Cary, NC: SAS Institute Inc.). Descriptive statistics were used for continuous variables, while frequencies and percentages were computed for categorical data. Association between variables were assessed using chi-squared or Fisher’s exact tests. A *p*-value of < 0.05 was taken to be significant.

### Ethical considerations

The study was approved by the Health Sciences Research Ethics Committee (HSREC) of the University of the Free State (ref. no. UFS-HSD2020/1481/2411). Permission to conduct the study was granted by the Head of the Free State Department of Health.

Following a detailed description of the study, signed informed consent was obtained from each participant prior to their participation in the study. The voluntary nature of participation and the right to refuse to participate or to withdraw at any time were also explained to the participants. The self-administered questionnaire was anonymous, as no identifying information was recorded on any of the documents.

## Results

### Sociodemographic and background characteristics of participants

Table 1 summarises the demographic data of the 341 participants. The median age of the participants at diagnosis was 66 years (range 40–93 years). Most patients were in their 70s (*n* = 162; 47.5%) while 68 (20.0%) were in their 50s.

**TABLE 1:** Sociodemographic and background characteristics of the participants (*n* = 341).

Variable	*n*	%
**Age at diagnosis (years)**		
40–49	4	1.2
50–59	68	20.0
60–69	162	47.5
≥ 70	107	31.3
**Cultural group**		
Sesotho	255	74.8
Tswana	54	15.8
Xhosa	22	6.4
Venda	6	1.8
Zulu	4	1.2
**Level of education**		
Some primary level (Grade 1–7)	141	41.3
Some secondary level (Grade 8–12)	90	26.4
Primary level (Grade 7) completed	45	13.2
No formal education	37	10.9
Grade 12 (matric)	25	7.3
Tertiary	3	0.9
**Relationship status**		
Married	255	74.8
Living as married or civil union	42	12.3
Widowed	26	7.6
Separated or divorced	12	3.5
Single or never married	6	1.8
**Level of skilled employment**		
Semi-skilled	165	48.4
Unskilled	140	41.1
Skilled	36	10.5
**Occupational exposure to mines (*n* = 103)**		
< 5 years	44	42.7
5–10 years	32	31.1
> 10 years	27	26.2
**Occupational exposure to herbicides or pesticides (*n* = 16)**		
< 5 years	15	93.7
5–10 years	0	0.0
> 10 years	1	6.3
**District or country of residence**		
Mangaung	162	47.5
Lejueleputswa	65	19.0
Thabo Mofutsayana	45	13.2
Fezile Dabi	34	10.0
Lesotho	16	4.7
Xhariep	15	4.4
Other	4	1.2
**Residential area**		
Rural	261	76.5
Urban	80	23.5

The majority of the participants (*n* = 298, 87.4%) had symptoms prior to the diagnosis of PCa. Of the 298 participants who had symptoms, 230 (77.2%) sought medical help themselves, and 67 (22.5%) were persuaded by family members, while one (0.3%) participant was advised on a PSA test by his doctor. The majority of the participants had multiple symptoms. The top 10 symptoms among the participants were dysuria (*n* = 216, 72.5%), poor stream (*n* = 203, 68.1%), urinary frequency (*n* = 129, 43.3%), nocturia (*n* = 98, 32.9%), urinary hesitancy (*n* = 78, 26.2%), frequent lower back pain (*n* = 75, 25.2%), impotence (*n* = 72, 24.2%), incomplete voiding (*n* = 47, 15.8%), dribbling of urine (*n* = 33, 11.1%) and urinary retention (*n* = 27, 9.1%).

Most patients presented to a healthcare facility within 1 year of symptoms (*n* = 238; 79.8%).

Only 76 (22.3%) participants had ever heard of PCa prior to diagnosis. Of these 76 participants, 36 (47.4%) had PCa screening in the past, by either PSA alone or in combination with digital rectal examination (DRE).

A total of 52 (15.2%) participants were aware of cancer history among first-degree family members.

A total of 226 (66.3%) participants had medical comorbidities, the most common being hypertension (*n* = 193, 85.4%), diabetes mellitus (*n* = 44, 19.5%), HIV infection (*n* = 22, 9.7%) and tuberculosis (*n* = 13, 5.8%).

A total of 89 (26.1%) participants had a past history of STDs; the majority (*n* = 55; 61.8%) of whom had just one episode, 33 (37.1%) had 2–5 episodes and one (1.1%) had > 5 episodes.

### Assessment of participants for environmental risk factors for prostate cancer

Table 2, Table 3 and Table 4 summarise the assessment of participants on environmental risk factors for PCa.

**TABLE 2:** Smoking, body size and exposure to sunlight.

Risk factor	Life stage (years)									
	20s (*n* = 341)		30s (*n* = 341)		40s (*n* = 341)		50s (*n* = 339)		≥ 60s (*n* = 302)	
	*n*	%	*n*	%	*n*	%	*n*	%	*n*	%
**Smoking (per day)**										
1 cigarette	1	0.3	1	0.3	3	0.9	1	0.3	0	0.0
2–5 cigarettes	31	9.1	28	8.2	31	9.1	31	9.1	26	8.6
6–10 cigarettes	82	24.0	86	25.2	81	23.8	64	18.9	31	10.3
11–20 cigarettes	47	13.8	48	14.1	43	12.6	30	8.8	16	5.3
> 20 cigarettes	2	0.6	2	0.6	1	0.3	0	0.0	0	0.0
Never smoked	178	52.2	173	50.7	172	50.4	170	50.1	150	49.7
Stopped smoking	0	0.0	3	0.9	10	2.9	43	12.7	79	26.1
**Body size estimate**										
Underweight	85	24.9	85	24.9	33	9.7	52	15.3	66	21.8
Normal weight	192	56.3	184	54.0	244	71.6	246	72.6	212	70.2
Overweight	60	17.6	68	19.9	61	17.9	37	10.9	20	6.6
Obese	4	1.2	4	1.2	3	0.9	4	1.2	4	1.3
**Exposure to sunlight**										
< 2 h	8	2.3	8	2.3	16	4.7	203	59.9	211	69.9
2 h – 5 h per week	181	53.1	182	53.4	212	62.2	115	33.9	75	24.8
6 h – 10 h per week	143	41.9	142	41.6	105	30.8	16	4.7	11	3.6
> 10 h per week	9	2.6	9	2.6	8	2.3	5	1.5	5	1.7

**TABLE 3:** Weekly physical activities and exercises.

Weekly physical activities and exercises	Life stage (years)									
	20s (*n* = 341)		30s (*n* = 341)		40s (*n* = 341)		50s (*n* = 339)		≥ 60s (*n* = 302)	
	*n*	%	*n*	%	*n*	%	*n*	%	*n*	%
**Walking**										
≤ 5 h	267	78.3	269	78.9	297	87.1	280	82.6	245	81.1
> 5 h	74	21.7	72	21.1	44	12.9	10	2.9	5	1.7
Never	0	0.0	0	0.0	0	0.0	49	14.5	52	17.2
**Home gardening**										
≤ 5 h	314	92.1	314	92.1	317	92.9	209	61.7	165	54.6
> 5 h	24	7.0	24	7.0	18	5.3	10	2.9	9	3.0
Never	3	0.9	3	0.9	6	1.8	120	35.4	128	42.4
**Gym**										
≤ 5 h	201	59.0	204	59.8	189	55.4	94	27.7	75	24.8
> 5 h	8	2.3	9	2.6	7	2.1	1	0.3	1	0.3
Never	132	38.7	128	37.5	145	42.5	244	72.0	226	74.8
**Housework**										
≤ 5 h	324	95.0	325	95.3	319	93.5	196	57.8	143	47.4
> 5 h	13	3.8	12	3.5	9	2.6	1	0.3	1	0.3
Never	4	1.2	4	1.2	13	3.8	142	41.9	158	52.3
**Social sport**										
≤ 5 h	270	79.2	268	78.6	226	66.3	68	20.0	53	17.5
> 5 h	8	2.3	8	2.3	4	1.2	0	0.0	0	0.0
Never	63	18.5	65	19.1	111	32.5	271	80.0	249	82.5

**TABLE 4:** Diet and eating habits.

Diet and eating habits	Life stage (years)									
	20s (*n* = 341)		30s (*n* = 341)		40s (*n* = 341)		50s (*n* = 339)		≥ 60s (*n* = 302)	
	*n*	%	*n*	%	*n*	%	*n*	%	*n*	%
**Carbohydrates**										
> 3 times per day	27	7.9	26	7.6	6	1.8	1	0.3	0	0.0
1–3 times per day	314	92.1	315	92.4	335	98.2	337	99.4	301	99.7
2–6 times per week	0	0.0	0	0.0	0	0.0	0	0.0	1	0.3
Once a week	0	0.0	0	0.0	0	0.0	1	0.3	0	0.0
< Once a week	0	0.0	0	0.0	0	0.0	0	0.0	0	0.0
**Dairy products**										
> 3 times per day	82	24.0	71	20.8	40	11.7	2	0.6	1	0.3
1–3 times per day	236	69.3	247	72.4	274	80.4	307	90.5	271	89.7
2–6 times per week	21	6.1	21	6.2	25	7.3	25	7.4	25	8.3
Once a week	1	0.3	1	0.3	1	0.3	4	1.2	4	1.3
< Once a week	1	0.3	1	0.3	1	0.3	1	0.3	1	0.3
**Fruits and vegetables**										
> 3 times per day	0	0.0	0	0.0	0	0.0	0	0.0	0	0.0
1–3 times per day	38	11.1	38	11.1	43	12.6	87	25.7	83	27.5
2–6 times per week	206	60.4	212	62.2	212	62.2	203	59.9	177	58.6
Once a week	74	21.7	68	20.0	67	19.6	48	14.1	41	13.6
< Once a week	23	6.7	23	6.7	19	5.6	1	0.3	1	0.3
**Red meat**										
> 3 times per day	0	0.0	0	0.0	0	0.0	0	0.0	0	0.0
1–3 times per day	132	38.7	132	38.7	134	39.3	123	36.3	105	34.7
2–6 times per week	198	56.1	198	56.1	197	57.8	201	59.3	182	60.3
Once a week	9	2.6	9	2.6	8	2.3	13	3.8	12	4.0
< Once a week	2	0.6	2	0.6	2	0.6	2	0.6	3	1.0
**Poultry**										
> 3 times per day	0	0.0	0	0.0	0	0.0	0	0.0	0	0.0
1–3 times per day	21	6.1	21	6.1	22	6.4	27	8.0	25	8.3
2–6 times per week	311	91.2	311	91.2	312	91.5	306	90.2	271	89.7
Once a week	5	1.5	5	1.5	5	1.5	5	1.5	5	1.7
< Once a week	4	1.2	4	1.2	2	0.6	1	0.3	1	0.3
**Fish**										
> 3 times per day	0	0.0	0	0.0	0	0.0	0	0.0	0	0.0
1–3 times per day	2	0.6	2	0.6	3	0.9	6	1.8	6	2.0
2–6 times per week	137	40.2	139	40.7	150	44.0	185	54.6	171	56.6
Once a week	149	43.7	148	43.4	145	42.5	128	37.7	112	37.1
< Once a week	50	14.6	49	14.4	39	11.4	17	5.0	11	3.6
Never	3	0.9	3	0.9	4	1.2	3	0.9	2	0.7
**Fast foods**										
> 3 times per day	0	0.0	0	0.0	0	0.0	0	0.0	0	0.0
1–3 times per day	0	0.0	0	0.0	0	0.0	1	0.3	1	0.3
2–6 times per week	12	3.5	13	3.8	40	11.7	45	13.3	28	9.3
Once a week	69	20.2	89	26.1	210	61.6	216	63.7	126	41.7
< Once a week	259	76.0	238	69.8	90	26.4	75	22.1	145	48.0
Never	1	0.3	1	0.3	1	0.3	2	0.6	2	0.7

At the time of the study, about a fifth (*n* = 76; 22.3%) of the participants were ≥ 6 years post diagnosis while the others (*n* = 265; 77.7%) were ≤ 5 years post diagnosis. The mean duration of PCa remission (at the time of the study) was 3.89 ± SD 3.21 years (range 1–17 years). Almost all (*n* = 319; 93.5%) of the participants had prostate biopsy and diagnosis within 3 months of referral to the urology unit. Likewise, the majority (*n* = 242; 71.0%) of the participants had commenced treatment within 3 months of diagnosis.

Table 5 summarises PCa staging and grading among the participants.

**TABLE 5:** Disease stage and grade at diagnosis among participants (*n* = 341).

Variable	*n*	%
**TNM staging**		
**Tumour (T)**		
T1	70	20.5
T2	138	40.5
T3	75	22.0
T4	58	17.0
**Lymph node (N)**		
N0	191	56.0
N1, N2, N3	43	12.6
NX (regional LN not assessed)	107	31.4
**Metastasis (M)**		
M0	197	57.8
M1	75	22.0
MX (unknown or cannot be evaluated)	69	20.2
**Diagnosis grade**		
**Gleason score**		
≤ 6 (grade group 1)	101	29.6
7 (3+4) (grade group 2)	91	26.7
7 (4+3) (grade group 3)	65	19.1
8 (grade group 4)	57	16.7
9 or 10 (grade group 5)	27	7.9

TNM, tumour, node, metastasis; LN, lymph node.

### Prostate cancer severity at diagnosis and associations with participants’ background characteristics and risk factors

The married participants (compared with the other relationship statuses) had heard about PCa (*p* = 0.034). Also, those with some secondary level education had heard about PCa (*p* < 0.001) and had previously screened for the disease (*p* = 0.001). Less than 10 years’ exposure to the mine was associated with the absence of lymph node metastasis (N0) (*p* = 0.020). Further associations between participants’ background characteristics and risk factors and PCa severity are summarised in Table 6.

**TABLE 6:** Associations between PCa severity at diagnosis and participants’ background characteristics and risk factors.

Variable	Outcome	*p*
Early presentation (≤ 6 months)	T1 or T2 tumour stage	< 0.001
	N0 (no nodal metastasis)	0.010
	M0 (no distant metastasis)	0.001
	Grade 1 or 2 disease	0.016
No PCa history in father	N0	0.016
	Grade 1 or 2 disease	0.028
No breast cancer history in mother	T1 tumour stage	0.002
	N0	0.018
	Grade 1 or 2 disease	0.006
Smoking ≥ 6 cigarettes per day	M1 (distant metastasis)	0.035
≤ 5 h per week sunlight exposure	T3 or T4 tumour stage	0.003
	M1	0.032
Less than 2 h walking per week	T3 or T4 tumour stage	0.019
Use of dairy products ≥ 2 times per day	T3 or T4 tumour stage	< 0.001
	Grade 4 or 5 disease	0.008
Consumption of fruits and vegetables 2–4 times per week	T1 tumour stage	< 0.001
	Grade 1 or 2 disease	0.009
Daily consumption of red meat	T3 or T4 tumour stage	< 0.001
	Grade 4 or 5 disease	0.007
	Nodal metastasis	0.005
	M1	0.033
2–6 times per week consumption of fish	T1	< 0.001
	Grade 1 or 2 disease	0.005
	N0	0.031
	M0	0.021

PCa, prostate cancer.

Symptomatic participants who sought medical attention within six months tended to have localised, non-metastatic and low-grade disease.

With regard to the history of PCa among first-degree family members, participants whose fathers did not have PCa tended to have absent nodal metastasis and low-grade disease. Also, those whose mothers did not have breast cancer had localised, non-metastatic (nodal) and low-grade disease.

Smoking ≥ 6 cigarettes per day was associated with metastatic disease.

A decreased (≤ 5 h per week) exposure to sunlight was associated with advanced and metastatic disease. Less than 2 h walk per week was associated with advanced disease.

The use of dairy products ≥ 2 times per day was associated with advanced and high-grade disease. The consumption of fruits and vegetables 2–4 times per week was associated with localised and low-grade disease. Daily consumption of red meat was associated with advanced, high-grade and metastatic disease. Eating fish 2–6 times per week was associated with localised, low-grade and non-metastatic disease.

## Discussion

### Background characteristics of participants

Age at diagnosis of PCa is one of the factors that contribute to poor health-related quality of life outcomes for survivors.^35^ In this study, the median age at diagnosis was 66 years. This is in keeping with statistics from the American Cancer Society, where 60% of cases are diagnosed in men who are 65 years or older and rare in men under 40 years.^36^ A recent retrospective study also found the average age at diagnosis to be 66 years among South African and Nigerian men.^37^ Early-stage PCa may be asymptomatic, and as such, most men will only present when symptomatic, especially if there is no PCa screening in the public sector. Hence, there is still the likelihood for delayed presentation in our setting.

Most men in this study are of the Sesotho cultural group. The ethnic differences shown in this study may be a reflection of ethnic group distribution in the Free State province and not necessarily an indication of the prevalence of the disease across the various ethnic groups. Studies have suggested that ancestry may be associated with PCa burden.^9,26,28^

Occupational exposure to carcinogenic agents such as pesticides, herbicides, chromium, cadmium, cutting fluids and ionising radiation is not uncommon in the Free State province, where mining and agriculture are the predominant industries. There is an association between exposure to these agents and PCa.^14,38^ Self-reported exposure to herbicides and pesticides in this study is relatively low (4.7%) compared with mine exposure (30.2%). This may be because of a lack of awareness of their exposure to hazardous occupational agents.

The majority (91.2%) of the participants had less than Grade 12 as their highest level of education. This may also explain why the majority (89.5%) were working in either unskilled or semiskilled jobs such as farming and agricultural work, mining, casual labour and other informal jobs.

### Clinical features among the participants

The majority (87.4%) of men in this study had LUTS prior to the diagnosis of PCa. It may therefore be useful for family physicians and GPs to consider symptomatic men for screening as recommended by SAPCDTG.^15^ Literature has described similar symptoms among patients, with 47% being asymptomatic. Bone ache and weight loss have been described as symptoms suggestive of metastatic diseases.^39^ Our study shows that about a quarter of the participants presented with lower back pain.

About 25% of the participants presented with impotence. This may however be because of androgen deficiency and certain cardiovascular diseases, which are not uncommon among the middle-aged and elderly. The top comorbid conditions among participants in this study were hypertension and diabetes mellitus.

Although the majority (77.2%) of the symptomatic men in this study sought medical attention out of self-conviction; the rest required some persuasion from family and healthcare providers. This may explain why more than 50% of the symptomatic men only sought medical attention after six months. According to Shaw et al.,^40^ involvement of family members in shared decision-making for PCa screening and treatment is often beneficial. In another study, the other reasons for delayed presentation include financial barriers, lack of health insurance and poor health-seeking behaviour.^9^

Less than a quarter (22.3%) of the participants had ever heard of PCa prior to diagnosis. This is in keeping with local^41^ and international^42,43^ studies that have shown poor knowledge and awareness of PCa among Black men. Almost half (47.4%) of the 76 participants who had earlier heard of PCa had ever screened for the disease; there may be the lack of screening opportunities in the public sector. Also, in a Kenyan study,^13^ despite massive education campaigns on PCa awareness, the screening rate was still low. Hence, apart from a knowledge gap, certain cultural factors were found to be responsible for the low turn-up for PCa screening.

### Risk factors for prostate cancer

Certain modifiable factors such as diet, lifestyle habits, infections and environmental exposure to chemicals or radiation, for PCa have been described.^9,14^

Positive associations between STDs have been described in studies.^44,45^ In our study, over a quarter (26.1%) of the participants reported past history of STDs.

Regarding nonmodifiable risk factors, about 15% of the participants gave a positive history of cancer among first-degree family members. Literature has shown that a history of PCa in a first-degree relative is associated with aggressive disease.^46^ Likewise, a history of female breast cancer in first-degree relatives was associated with an increased risk of PCa, often of a high grade.^47^

### Stage and grade of prostate cancer among participants

In this study, 39.0% of the participants presented with T stage ≥ T3, 22.0% presented with metastatic disease, and 24.6% had Gleason score ≥ 8. In a similar local study,^48^ 62.3% had T stage ≥ T3, and 43.7% had a Gleason score ≥ 8. This above-mentioned study included men of other races, and only participants on treatment were included; this may explain the differences in the stage and grade of the disease.

### Prostate cancer stage and grade at diagnosis and associations with participants’ background characteristics and risk factors

Marital status has been shown to be an important factor associated with PCa stage and grade at diagnosis. In a study on marital status and PCa incidence,^49^ widowers were shown to have worse cancer stage at diagnosis. Although there was no association of statistical significance between marital status and disease grade in this study, married participants were more likely to be aware of PCa (*p* = 0.034).

Level of education and health literacy are risk factors for a higher stage of PCa at diagnosis.^50^ Although there was no association of statistical significance between education level and disease grade in this study, participants with at least a secondary level education were more aware of the disease (*p* < 0.001) and were more likely to have been previously screened for the disease (*p* = 0.001).

The earlier the diagnosis, the better the prognosis. Worse prognosis has been shown where there is a decreased awareness of the disease and late presentation.^14^ Our study showed that symptomatic men who presented earlier (within 6 months) were more likely to have T1 stage (*p* < 0.001), low grade disease, that is, Gleason grade 1 (*p* = 0.016), absence of lymph node metastasis (*p* = 0.010) and absent distant metastasis (*p* = 0.001).

A study conducted in the United States showed an association between exposure to cadmium and aggressiveness of PCa.^51^ In our study, participants with less than 10 years mine exposure were more likely to present with PCa without lymph node metastasis (*p* = 0.020).

Participants with a negative history of PCa among first-degree family members were more likely to present with low grade disease, that is, Gleason grade 1 (*p* = 0.028) and absent lymph node metastasis (*p* = 0.016). Those with negative history of breast cancer among first-degree family members were more likely to have T1 stage (*p* = 0.002), low grade disease, that is, Gleason grade 1 (*p* = 0.006) and absent lymph node metastasis (*p* = 0.018).

Certain smoking patterns (onset, intensity and frequency) have been shown to be associated with higher poorly differentiated PCa.^52,53,54^ Just over half of the participants in our study had never smoked cigarettes. Of the group that smoked, the majority smoked 6–10 cigarettes daily. Smoking ≥ 6 cigarettes per day was associated with metastatic PCa (*p* = 0.035).

Vitamin D deficiency has been associated with certain cancers, including PCa.^55^ Sunlight exposure is a vital process in producing vitamin D3 in the skin from 7-dehydrocholesterol, which is metabolised in the liver and kidney into the active form.^56^ Sun exposure in early life has been shown to protect against PCa. Frequent sun exposure in adulthood has been shown to be associated with a significantly reduced risk of fatal PCa.^57^ As shown in Table 2, the majority of the participants younger than 50 years report 2 h – 5 h of weekly exposure to sunlight. Participants in their 50s and older mainly reported less than 2 h sun exposure per week. A decreased exposure to sunlight was associated with T3 or T4 tumour stage (*p* = 0.003) and metastatic disease (*p* = 0.032).

Physical activity helps to decrease the deposition of central adipose tissue. It also lessens circulating levels of inflammation, insulin and unfavourable sex hormones, thereby preventing PCa progression.^58^ As seen on Table 3, the majority of the participants engaged in weekly physical activities of ≤ 5 h across the life phases. Less than 2 h walk per week was associated with T3 or T4 tumour stage (*p* = 0.019).

Certain diets or eating habits have been associated with an increased risk of developing PCa.^14,54^ The staple foods in most Southern African nations are corn, wheat-based and dairy products. Also, agriculture and farming are among the prevalent industries in the Free State province; these may therefore explain the reason for the majority of the participants consuming carbohydrates (96.4%) and dairy products (80.5%) 1–3 times daily. Certain cultures believe a meal is incomplete without meat. Also, as earlier stated, the province is notable for agriculture and farming; hence, there may be a relative increased access to these food products, including fruits and vegetables. As shown in Table 4, the majority of the participants consumed red meat (57.9%), poultry (90.8%), fish (47.2%), fruits and vegetables (60.7%) 2–6 times per week. The majority (72.9%) of participants in their 20s and 30s ate fast food less than once a week. This may be because of a lack of affordability. The frequency of consuming fast food, however, increased to once a week (55.7%) in those over 40 years of age.

Eating fruits and vegetables 2–4 times per week was associated with T1 tumour stage (*p* < 0.001) and grade 1 or 2 disease (*p* = 0.009). Also, eating fish 2–6 times per week was associated with T1 tumour stage (*p* < 0.001), grade 1 or 2 disease (*p* = 0.005), absent lymph node metastasis (*p* = 0.031) and absent distant metastasis (*p* = 0.021). On the contrary, daily consumption of red meat was associated with T3 or T4 tumour stage (*p* < 0.001), grade 4 or 5 disease (*p* = 0.007), lymph node metastasis (*p* = 0.005) and distant metastasis (*p* = 0.033). Also, the use of dairy products ≥ 2 times per day was associated with T3 or T4 tumour stage (*p* < 0.001) and grade 4 or 5 disease (*p* = 0.008).

While treatment delay of several months or even years may not affect outcomes of men with low-risk PCa, the same cannot be said when the PCa is not low-risk.^59^ In a study among patients who underwent radical prostatectomy, a surgical delay time of up to six months after diagnosis was not associated with higher risks of having any adverse pathological outcomes or worse overall survival.^60^ In another study^61^ among patients who underwent low-dose-rate brachytherapy, treatment delay of more than six months appeared to adversely correlate with biochemical recurrence-free survival. Therefore, it was suggested that even low- and intermediate-risk PCa patients should have brachytherapy performed within six months of the diagnosis.^61^

Our study showed that most (93.5%) of the participants had histological confirmation of the disease within three months of referral from a primary health care facility. Also, the majority (98.8%) had commenced treatment within six months of diagnosis. Therefore, there seem to be no significant systems delays in the diagnostic and therapeutic process relating to PCa at the higher healthcare facilities of the Free State province.

### Strengths and limitations

As far as we know, this is the first study in the study setting focusing on the more vulnerable group, that is, men of African descent. However, several limitations of this study should be noted. Firstly, certain relevant information was absent in the patients’ case files, making it mandatory to interview live subjects; the use of case files (alone) would have increased the sample size, giving more credence to the study. However, all possible subjects were included in the study and data collection continued until data saturation was reached. Secondly, this was a cross-sectional descriptive study; therefore, a cause–effect relationship cannot be claimed. Lastly, with a median age of 66 years among the participants, recall bias was possible.

## Conclusion and recommendations

Late-stage (T3 or T4), poor grade (Gleason ≥ 8) and metastatic PCa disease are not uncommon among men ≥ 60 years in our study setting. The majority had LUTS prior to diagnosis but were of poor health-seeking behaviour. Certain modifiable risk factors associated with advanced disease such as smoking, decreased sunlight exposure, decreased physical activity and increased ingestion of red meat and dairy products were established in this study. Despite poor awareness of the participants prior to PCa diagnosis, once diagnosed, there was no delay in treatment. Because of the high prevalence of advanced and high-grade PCa disease and the possible associated modifiable risk factors along with poor awareness of the disease, a prompt community-specific health promotion strategy is needed. Also, targeted PSA screening should be considered among men with nonmodifiable risk factors and even more promptly in the presence of LUTS.
